# Coagulation tests or standardized questionnaire, which is better as a predictor of bleeding? A prospective study among children before tonsillectomy and/or adenoidectomy

**DOI:** 10.1186/s13104-020-05020-6

**Published:** 2020-03-24

**Authors:** Muhamed Masalha, Ari DeRowe, Salim Mazzawi, Tzvi Chen, Rami Ghanayim, Roee Landsberg, Ariel Koren

**Affiliations:** 1 Deptartment of Otolaryngology Head and Neck Surgery, Emek Medical Center (affiliated with The Ruth and Bruce Rappaport School of Medicine, Technion Institute of Technology, Haifa), Yitzhak Rabin Boulevard 21, 1834111 Afula, Israel; 2 Deptartment of Otolaryngology Head and Neck Surgery, Pediatric Otolaryngology, Tel Aviv Sourasky Medical Center (affiliated with Sackler School of Medicine, Tel Aviv University), 6 Weizmann Street, 64239 Tel Aviv, Israel; 3 Otolaryngology, Head and Neck Surgery, Assuta Medical Center (affiliated with Ben Gurion University-Faculty of Medicine, Beer Sheva), 20 Habarzel St, Ramat Hachayal, 69710 Tel Aviv, Israel; 4 Pediatric Hematology Unit, Emek Medical Centre (affiliated with The Ruth and Bruce Rappaport School of Medicine, Technion Institute of Technology, Haifa), Yitzhak Rabin Boulevard 21, 1834111 Afula, Israel

**Keywords:** Bleeding, Coagulation tests, Tonsillectomy, Adenoidectomy, Questionnaire

## Abstract

**Objective:**

The value of pre-operative coagulation testing for adenotonsillar surgery is controversial. The purpose of this study was to evaluate the role of routine coagulation tests and a standardized questionnaire in children before tonsillectomy and/or adenoidectomy.

**Results:**

A total of 143 children were prospectively enrolled in the study between 2013 and 2017, 81 males (56.6%) and 62 females (43.4%), age range 1 to 18 years (median age 5 years). Eighteen bleeding events were documented, three of them required treatment in the operating room. Abnormal coagulation tests were not associated with higher odds of bleeding after surgery. Higher risk of bleeding (p = 0.01) was associated with an abnormal standardized medical questionnaire.

## Introduction

Tonsillectomy and/or adenoidectomy (T/AD) is a frequently performed procedure in children. 300,000 surgeries are carried out annually in the United States [1]. The main indications for T/AD are obstructive sleep disturbances (mainly in younger children), and recurrent tonsillitis (often in older children) [2].

In T/AD surgery the tonsillar tissue is dissected in the plane between the tonsillar capsule and the pharyngeal muscles. This can be performed with different techniques; cold dissection, monopolar or bipolar electrocautery, radiofrequency and coblation [3].

Bleeding is probably the most important, serious, and discussed complication associated with T/AD. The risk for post T/AD bleeding is 1–5.7 percent by some reports [4] and 2–7% by others [5, 6]. Some studies report even higher and wider range [7, 8]. Immediate post-operative bleeding, occurring in the first 24 h after surgery, can be attributed to inadequate operative hemostasis [1]. Delayed bleeding, occurring later is thought to be the result of sloughing a covering eschar or loosened vessel tie [1, 7].

In candidates for T/AD there is a consensus for screening for a personal and familial history of bleeding and is typically performed during pre-operative evaluation. However, the utility of pre-operative routine coagulation tests is controversial [1]. Both the British Committee for standards in Hematology 2008 and the American Academy of Otolaryngology-Head and Neck surgery did not recommend standard pre-operative coagulation tests [1, 9]. Recommendations against performing standardized coagulation laboratory test are supported by a large body of literature [4–6, 9, 10]. However, several articles show a contradicting opinion, suggesting that performing those tests pre-operatively may be beneficial in predicting potential bleeding and coagulopathies [1, 7, 11, 12].

Preoperative bleeding history questionnaires (POBQ) have been developed by several researchers [4]. Such questionnaires aim to standardize the pre-operative patients’ history evaluation before T/AD, avoiding the loss of important data that may predict bleeding tendency and to improve uniformity of the pre-operative evaluation process among caregivers.

In this study we propose to further determine the need for pre-operative coagulation tests before T/AD surgery in children. We also try to evaluate the value of a standardized pre-operative questionnaire, alone or with routine coagulation tests in predicting the risk to bleed after T/AD.

## Main text

### Methods

Children of both genders, less than 18 years of age who had been scheduled for T/AD between January 2013 and December 2017 at Emek Medical Center were included prospectively. One of their parents or legal custodians signed informed consent. Only patients who underwent surgery were included in this study.

The main indications for surgery were obstructive sleep disturbances or recurrent tonsillitis. Ages ranged from 1 to 18 years. A recent pre-operative platelet counts and routine blood coagulation tests including prothrombin time (PT), PT ratio, activated partial thromboplastin time (aPTT), aPTT ratio and PT international normalized ratio (INR) results were reviewed according to the routine protocols used in our department. Abnormal results were defined as any result falling beyond the normal scale values; for aPTT a prolonged time above 37″, for INR above 1.2, for PT above 13.6″ and for platelets count below 150,000× 10^9^/dl.

A standardized pre-operative bleeding history questionnaire was given to every participant parent. The questionnaire consisted of three groups of questions; the first group dealt with the child’s personal history of bleeding, the second with family history, and the third group of questions dealt with general medication history. Any positive response in any of the above mentioned parts was defined as a positive questionnaire.

All abnormal coagulation studies were reviewed by the pediatric hematologist before approving the surgery. After reviewing children with positive questionnaire we decided to operate them without further evaluation.

All patients underwent surgery using the same technique, monopolar diathermy.

At least 2 weeks after surgery, patients’ parents were called, and a second questionnaire was completed during this phone call. The second questionnaire reviewed the followings issues: bleeding after surgery, time of bleeding, and specific treatment given. Post-operative bleeding was defined by any amount of fresh nasal or oral blood after surgery observed by a caregiver.

Data was collected and summarized, with continuous variables presented as means and standard deviation along with median and range, and categorical data as number and percent. Comparison of the two groups was made by Chi square or Fisher’s exact test where appropriate for categorical data and by the Mann–Whitney test for continuous data. Relative risk (RR) ratio and 95% confidence interval (CI) were computed to assess the rate of bleeding. Statistical significance was set at p (probability value) < 0.05. Data analysis was performed using SPSS version 21 and Winpepi.

### Results

A total of 143 children were enrolled in the study, 81 males (56.6%) and 62 females (43.4%). Age ranged between 1 and 18 years with a mean age of 5.6 ± 3.4 years old [median = 5 years].

Eighteen out of 143 children were documented to have bleeding after surgery (12.6%). Three of them required treatment in the operating room. On average the bleeding occurred after 4.9 ± 5.9 days [median = 4 days]. Ten of the bleeders had mild bleeding that stopped spontaneously at home and they either didn’t receive any medical care or were reassured by a primary physician. Eight children were examined in the emergency room and in five of these cases the bleeding stopped spontaneously; all these children were admitted for observation and released in good condition the following day. The remaining three children (2.1%) underwent surgical intervention to control bleeding and were released after a short period of observation in hospital.

Out of 143 patients, 111 (81.0%) had normal coagulation screening tests and 26 (19.0%) had abnormal results. In 6 children coagulation screening tests were not available. However, the tests were reported to be normal by a parent or by a primary doctor. Eighteen patients bled after surgery, with 13 (11.7%) having normal coagulation screening tests and five (19.2%) abnormal screening test (four had mild prolonged aPTT ranged from 37.4″ to 38.7″ and one had a mild abnormal PT of 13.7″). The bleeding rate between patients having a normal or abnormal coagulation screening test was not statistically significant (χ^2^ = 1.04, Fisher’s exact p = 0.34, RR:1.09; 95% CI 0.90–1.33; Table 1). Table 2 presents the results of the coagulation tests. There were no statistically significant differences between these tests in children who bled and those who did not bleed although the children who did not bleed tended to have higher platelet count than their counterparts (Z = 1.73 p < 0.08).

**Table 1:** The impact of abnormal coagulation tests on bleeding

Risk factor	Total (N = 137) N (%)	Bleeding (N = 18) N (%)	No Bleeding (N = 119) N (%)	p	RR (95% CI)
PTT > 37″ s	19 (14.6)	4 (21.1)	15 (78.9)	0.27	1.76
PTT ≤ 37″ s	111 (85.4)	14 (12.6)	97 (87.4)		(0.66–4.72)
INR above 1.2	0 (0.0)	0 (0.0)	0 (0.0)	–	–
PT > 13.6″ s	6 (4.6)	1 (16.7)	5 (83.3)	> 0.99	1.04
PT ≤ 13.6″ s	125 (95.4)	17 (13.6)	114 (86.4)		(0.72–1.49)
Plt < 150 × 10^9^/dl	1 (0.7)	0 (0.0)	1 (100.0)	> 0.99	0.42*
Plt ≥ 150 × 10^9^/dl	136 (99.3)	16 (11.8)	118 (88.2)		(0.02–7.45)
Abn results	26 (19.0)	5 (19.2)	21 (80.8)	0.34	1.09
Normal results	111 (81.0)	13 (11.7)	98 (88.3)		(0.90–1.33)

*N* number, *RR* risk ratio, *CI* confidence interval, *Plt* platlets, *Abn* abnormal

*Adjusted ratio with 0.5 added to each cell

**Table 2:** Coagulation tests of children who bled vs. children who didn’t

	Group		p
	Bleeding (N = 18) N (%)	Non bleeding (N = 125) N (%)	
Abnormal coagulation tests results	5 (19.2%)	21 (80.8%)	0.34
Normal test results	13 (11.1%)	104 (88.9%)	
	Mean ± SD [median, range]	Mean ± SD [median, range]	
Platelets 10^9^/dl	301.06 ± 73.97 [294.5, 196–453]	338.24 ± 86.73 [335.5, 132–764]	0.08
PTT Sec	33.68 ± 3.62 [33.9, 26.7–38.7]	332.098 ± 3.782 [32.87, 24.7–46.8]	0.34
aPTT ratio	1.19 ± 0.12 [1.20, 0.99–1.39]	1.187 ± 0.13 [1.1728, 0.89–1.68]	0.63
PT Sec	11.96 ± 0.96 [12.15, 10.1–13.7]	11.7495 ± 12.0142 [11.60, 9.9–135.32]	0.19
PT (%)	96.61 ± 11.20 [93.5, 80–121]	100.945 ± 11.05 [100, 82–130]	0.12
INR	1.01 ± 0.07 [1.03, 0.88–1.14]	0.99 ± 0.07 [0.99, 0.84–1.15]	0.20

*N* number, *SD* standard deviation, *p* probability value

Out of 143 children, 20 (14.0%) had abnormal positive questionnaire during the pre-operative evaluation. Six of those children suffered from a bleeding event (30.0%). In the group of 123 (86.0%) children with a negative questionnaire, there were 12 (9.8%) bleeders. This difference was statistically significant (χ^2^ = 6.41, p = 0.011) (Table 1). The rate of a child who had an abnormal positive questionnaire during the pre-operative evaluation to bleed were more than 3 times that of children who had a normal questionnaire (RR: 3.08; 95% CI 1.30–7.26).

In only four patients both the pre-operative coagulation tests and the questionnaire were abnormal. Of them two patients (50%) suffered from a bleeding event. When both the questionnaire and the coagulation tests were abnormal, the risk of bleeding was not found to be statistically significantly higher. (χ^2^ = 5.25, Fisher’s exact p = 0.08; RR: 6.94; 95% CI 1.04–46.27) (Table 3).

**Table 3:** Comparison between the bleeding group and the non-bleeding group

	Bleeding (N = 18)	No bleeding (N = 125)	p value	RR (95% CI)
Questionnaire positive	6 (33.3%)	14 (11.2%)	0.01	3.08 (1.30–7.26)
Questionnaire & coagulation abnormal	2 (11.1%)	2 (1.6%)	0.08	6.94 (1.04–46.27)
Gender				
Male	13 (72.2%)	68 (54.4%)	0.15	1.33 (0.96–1.84)
Female	5 (27.8%)	57 (45.6%)		

	Mean ± SD [median; range]	Mean ± SD [median; range]		
Age (years)	6.9 ± 4.3 [5.5; 3–18]	5.4 ± 3.2 [5.0; 1–17]	0.25	1.11* (0.98–1.26)

*N* number, *RR* risk ratio, *CI* confidence interval, *AD* adenoidectomy, *TON* tonsillectomy, *SD* standard deviation

*Odds ratio estimate of RR

Out of the three patients that required intervention in the operating room for bleeding control, one patient had both pre-operative abnormal coagulation tests (only mild prolonged PT″ of 13.7) and positive questionnaire. The other two patients had normal pre-operative evaluation.

### Discussion

The preoperative evaluation before T/AD surgeries is important and should emphasize bleeding risks.

When we compared, qualitatively and quantitatively, the coagulation tests’ results between the group of bleeders and non-bleeders, neither abnormal results nor the value of the results showed a significant difference between the groups (Tables 1 and 2). The rate of bleeders in patients with normal coagulation tests was 11.6%, while in the group with abnormal blood tests was 19.2%, these higher rates in the bleeding group were not statistically significant, but can still give a feeling that coagulation tests though not sensitive in predicting bleeding, may have additional value and should not be initially invalidated. We agree with the opinion of many researchers that it may not be necessary to perform these tests in every child prior to T/AD [4–6, 9, 10], however, we recommend these tests in any child with a positive medical history or questionnaire. When both the questionnaire and the coagulation tests are positive, pediatric hematologist consultation should be considered. Coagulation tests may further ascertain the normal risk for bleeding in some cases and may unveil some hidden conditions that are worth managing before surgery. Children with complex medical conditions such as obesity, and craniofacial abnormalities or syndromes, are to be considered for performing coagulation tests before surgery. These children are at increased risk for surgical or anesthetic complications [13], and any further post-operative re-intervention may carry a higher general risk.

Seeking relevant personal and family history prior to T/AD surgery is widely accepted. In our study, obtaining medical history before surgery, using standardized questionnaire, could predict higher risk for bleeding after T/AD surgeries in children, therefore, further evaluation of these children should be considered. Standardization of medical history using a dedicated questionnaire has the advantage of standardizing the assessment process among various medical personnel and avoids omitting portions of the relevant history. Our impression is that both the caregivers and the patients’ family are comfortable using this questionnaire, in a relatively short time and with ease. This tool is concise, cheap, noninvasive, and facilitates acquiring a complete history and physical examination.

Four children had both abnormal coagulation test and abnormal questionnaire, two of them (50%) bled (p = 0.08, Table 3) and one required treatment in the operating room. Although not statistically significant, this could be due to a small sample size. Probably, a combined abnormal questionnaire along with abnormal coagulation tests improves the ability to predict the risk to bleed better than each alone, we recommend hematological consultation for these patients before surgery.

The overall bleeding rate in this study was 12.5%. The reported rate in the literature is usually between 2 and 7% [5, 6], other reports suggest a wider range of up to 20% [7, 8]. At a glance, it seems that the bleeding rate in our study is higher than that reported, but other factors should be taken into consideration in this analysis. Firstly, it is a prospective study with a fixed post-operative follow-up; every bleeding event, even though very minor is reported and documented. In our study, in most children who were reported as bleeders (10 out of 18), the bleeding had stopped spontaneously and they were followed at home. Five children presented to the emergency room and were admitted to hospital for observation with no intervention. Only 3 patients (2.1%) returned to the operating room for control hemorrhage. This rate is consistent with the literature. Secondly, there is a wide variation in the reported rate of bleeding in literature (2 to 20%), which arises mainly from different size and age structures of patient’s population, various indications for surgery, studies type, and different duration of post-operative follow up [14]. Therefore, the bleeding rate in our study is acceptable.

In conclusion, in this study we show that routine pre-operative coagulation tests before T/AD surgeries in children is not necessary and should be reserved for special risk population. Pre-operative abnormal standardized medical questionnaire was found to accurately predict children with higher risk to bleed after T/AD surgeries and should routinely be used in the evaluation process before surgery. When the questionnaire reveals a risk, further workup including coagulation tests and/or pediatric hematology consultation should be considered.

## Limitations

One of our major limitations in this study was the small populations size of the children who bled, of the children with abnormal questionnaire and or abnormal coagulation tests, which affected our ability to accurately reflect significance. Moreover, it would be beneficial if we had performed complete coagulation tests analysis including clotting factors analysis for every child with abnormal questionnaire, something that we could not do in this study and should be addressed in future studies.

## Data Availability

The datasets used and/or analysed during the current study available from the corresponding author on reasonable request.

## Abbreviations

T/AD: Tonsillectomy and/or adenoidectomy
POBQ: Preoperative bleeding history questionnaires
PT: Prothrombin time
aPTT: Activated partial thromboplastin time
INR: PT international normalized ratio
RR: relative risk
CI: Confidence interval
p: Probability value
